# Accuracy of artificial intelligence-assisted endoscopy in the diagnosis of gastric intestinal metaplasia: A systematic review and meta-analysis

**DOI:** 10.1371/journal.pone.0303421

**Published:** 2024-05-14

**Authors:** Na Li, Jian Yang, Xiaodong Li, Yanting Shi, Kunhong Wang

**Affiliations:** Department of Gastroenterology, Zibo Central Hospital, Zibo, Shandong, China; Dalin Tzu Chi Hospital, Buddhist Tzu Chi Medical Foundation, TAIWAN

## Abstract

**Background and aims:**

Gastric intestinal metaplasia is a precancerous disease, and a timely diagnosis is essential to delay or halt cancer progression. Artificial intelligence (AI) has found widespread application in the field of disease diagnosis. This study aimed to conduct a comprehensive evaluation of AI’s diagnostic accuracy in detecting gastric intestinal metaplasia in endoscopy, compare it to endoscopists’ ability, and explore the main factors affecting AI’s performance.

**Methods:**

The study followed the PRISMA-DTA guidelines, and the PubMed, Embase, Web of Science, Cochrane, and IEEE Xplore databases were searched to include relevant studies published by October 2023. We extracted the key features and experimental data of each study and combined the sensitivity and specificity metrics by meta-analysis. We then compared the diagnostic ability of the AI versus the endoscopists using the same test data.

**Results:**

Twelve studies with 11,173 patients were included, demonstrating AI models’ efficacy in diagnosing gastric intestinal metaplasia. The meta-analysis yielded a pooled sensitivity of 94% (95% confidence interval: 0.92–0.96) and specificity of 93% (95% confidence interval: 0.89–0.95). The combined area under the receiver operating characteristics curve was 0.97. The results of meta-regression and subgroup analysis showed that factors such as study design, endoscopy type, number of training images, and algorithm had a significant effect on the diagnostic performance of AI. The AI exhibited a higher diagnostic capacity than endoscopists (sensitivity: 95% vs. 79%).

**Conclusions:**

AI-aided diagnosis of gastric intestinal metaplasia using endoscopy showed high performance and clinical diagnostic value. However, further prospective studies are required to validate these findings.

## Introduction

Gastric cancer ranks fifth in terms of global cancer prevalence, posing a serious threat to human health [1]. Although the incidence of gastric cancer has decreased over the past three decades, the absolute number of cases continues to rise due to an aging population and a shift towards younger age groups developing gastric cancer. Hence, reducing the incidence and mortality of gastric cancer remains an urgent issue [1].

The progression of the most gastric cancers is a cascade pattern, which includes gastritis, atrophic gastritis (AG), intestinal metaplasia, heterogeneous hyperplasia, lastly culminating in cancer [2, 3]. AG and gastric intestinal metaplasia (GIM) are important intermediate- and high-risk factors for the development of gastric cancer. Early detection of these lesions is essential for delaying or halting the development of gastric cancer. In clinical practice, white light endoscopy is typically used to observe gastric lesions. However, studies have shown that the correlation between histology and general white light endoscopy diagnosis is low [4–8].

In the last decade, artificial intelligence (AI) has garnered significant attention within the scientific community, leading to considerable research being conducted on AI-related subjects, such as neural networks, machine learning, and deep learning. It has been used in various industries to provide powerful solutions to complex problems [9–11]. Computer vision is an important research area in AI. By applying various algorithms, computer vision systems can analyze and extract meaningful information from images or videos. Image classification algorithms are used to identify the category to which an image belongs, represented by VGG [12], ResNet [13], TResNet [14], SE-ResNet [15], and EfficientNet [16]. Object detection algorithms focus on finding one or more targets in an image and framing them with rectangular boxes; typical algorithms are SSD [17], YOLO [18, 19], and R-CNN [20]. Semantic segmentation algorithms identify each pixel in the image and is capable of accurate segmentation based on the boundary of the target; typical algorithms are UNet++ [21], DeepLab [22, 23], and BiSeNet [24]. These techniques are widely used in medical imaging diagnoses [25, 26].

In gastrointestinal endoscopy, AI has been used to diagnose various diseases [27–29] and has achieved good diagnostic efficacy. Bang et al. [30] performed a meta-analysis including eight studies that specifically examined the accuracy of AI-assisted endoscopy in the diagnosis of *Helicobacter pylori* infection. Our previous study [31] conducted a meta-analysis on the accuracy of AI-assisted endoscopy in diagnosing chronic atrophic gastritis. In this study, we utilized meta-analysis to evaluate the accuracy of AI in diagnosing GIM, explored the main factors affecting AI’s ability, and compared AI performance with that of endoscopists, thereby providing an objective basis for applying AI in clinical diagnosis.

## Methods

Before commencing the study, we had registered it with PROSPERO [32] (ID: CRD42022378974). This study strictly followed the PRISMA-DTA [33] guidelines. The associated checklist for PRISMA-DTA can be found in S1 Table.

### Searching strategy

To obtain relevant studies, we searched the following five databases from their establishment up to October 2023: PubMed, Embase, Web of Science, Cochrane Library, and IEEE Xplore. Notably, PubMed, Embase and Cochrane Library are common medical databases, while the Web of Science is an extensive and comprehensive database. The IEEE Xplore database covers computer science, electronics, and other related fields. Related search terms include: artificial intelligence, deep learning, machine learning, computer-aided diagnosis, neural networks, gastritis, gastric precancerous, gastric tissue, and intestinal metaplasia. The detailed search strategy is shown in S2 Table.

### Study selection

Inclusion criteria: (a) Studies use AI technology to analyze endoscopic images/videos to detect GIM lesions. (b) Ability to extract 2x2 table data from articles. (c) Clear presentation of diagnostic criteria. (d) A clear description of the AI algorithm and the process of diagnosing GIM. (e) The most recent studies from multiple studies on the same research group, if the AI model or study cohort was the same. Exclusion criteria: (a) Studies in which the full text was unavailable. (b) Studies in which complete four-grid table data were unavailable. (c) Reviews, meta-analyses, editorial reviews, letters to the editor, conference abstracts, and other types of literature. Two authors (J.Y. and X.L.) independently evaluated the search results, and any disagreements were resolved through discussion.

### Data extraction

The key data we extracted from each study included the first author, publication year, country/region, study design, study center, diagnostic criteria, algorithm, number of training set samples, test set type, number of test set samples, and 2x2 table data. Two authors (Y.S. and X.L.) independently extracted the data by reading the full text, and disagreements were resolved through discussion.

### Quality assessment

QUADAS-2 [34] is the widely used quality assessment tool for diagnostic accuracy studies, and includes four parts: Patient Selection, Index Testing, Reference Standards, and Flow and Timing. However, QUADAS-2 is not fully applicable to AI-centered diagnostic accuracy studies [35, 36]; therefore, we supplemented QUADAS-2 to make it more suitable for AI-centered studies. In the patient selection section, the source, size, and quality of the input data were accurately described. In index testing, whether the AI model is tested using an independent test set. In the reference standard section, whether pathological tissue biopsies were used as the “Gold Standard” is described.

### Statistical analysis

Based on a bivariate mixed-effects model, we calculated diagnostic performance indicators such as combined sensitivity, specificity, and diagnostic odds ratio (DOR). The likelihood ratio is a composite index that reflects sensitivity and specificity. The positive likelihood ratio (PLR) > 10 and the negative likelihood ratio (NLR) < 0.1 indicate high diagnostic performance. The area under the curve (AUC) and DOR are comprehensive measures to evaluate diagnostic accuracy. AUC> = 0.9 indicates the high accuracy of the diagnostic test. A larger DOR value indicated a better diagnostic performance.

The heterogeneity of the studies was assessed by the visual inspection of summary receiver operating characteristic and forest plots and counted by the I^2^ value. If heterogeneity existed, a subgroup analysis and meta-regression were performed. The clinical applicability was assessed using Fagan plots. Deek’s funnel plot assessed publication bias, and when the angle between the straight line in the plot and the coordinate X-axis was closer to 90°, it indicated the existence of publication bias. When P<0.05 was statistically significant, publication bias was present.

Quality assessment of the included studies was performed using Review Manager 5.4 (Cochrane Collaboration, Oxford, UK). Other statistical analyses and graphing were conducted using Stata/SE16.0 (Stata, TX, USA).

## Result

### Included studies

**Fig 1:**
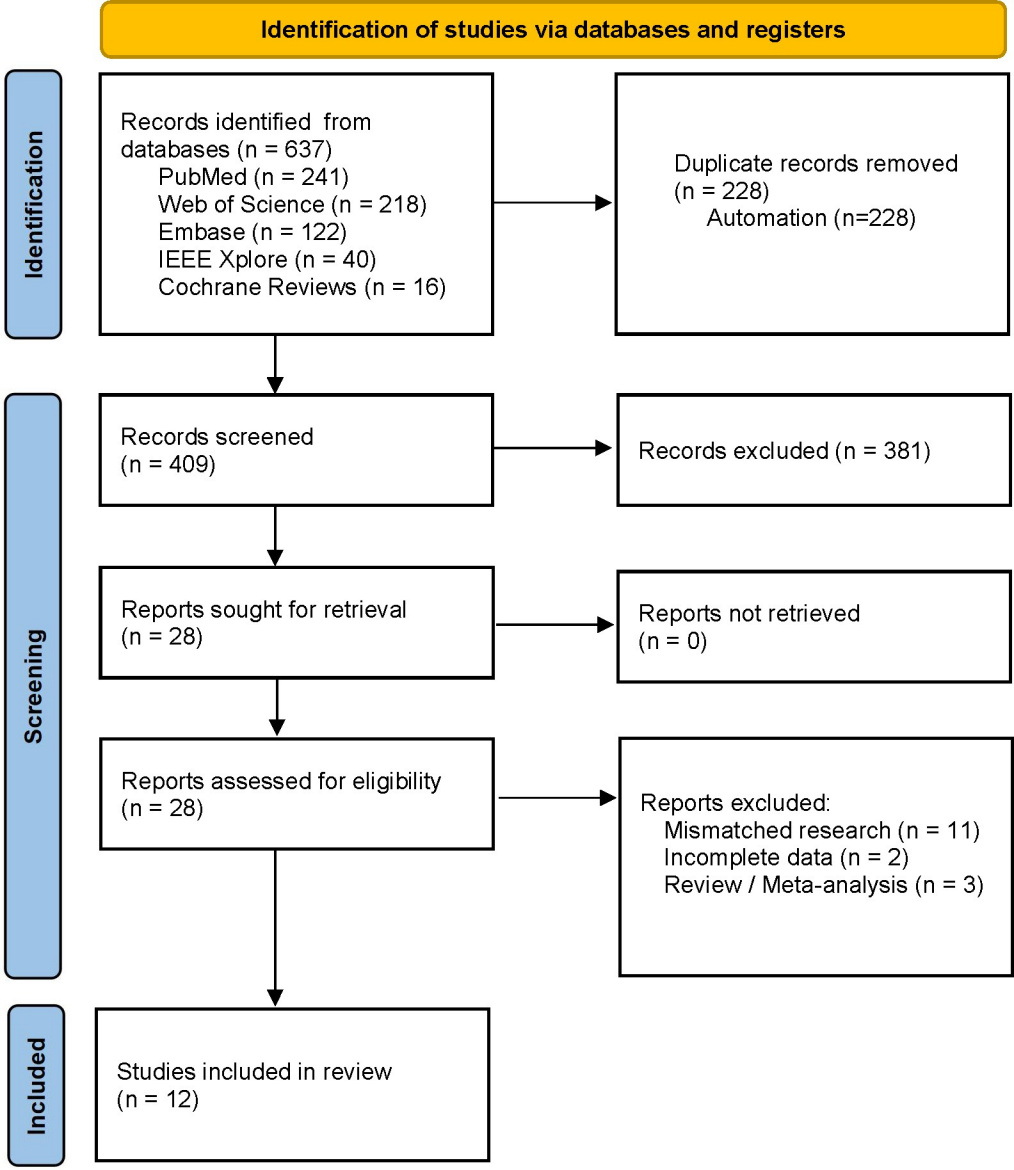
Flow diagram for study selection.

The final search was conducted on October 12, 2023, yielding 637 papers. Among these, 228 duplicates were automatically removed through EndNote, 381 irrelevant papers were excluded by reading the titles and abstracts, two were excluded without retrieving the full text, and 14 were excluded after examining the full text. Twelve studies [37–48] (Table 1) were finally included. The flow diagram for study selection is shown in Fig 1.

**Table 1:** Included studies.

Study	Country/Region	Endoscopy	Algorithm	Study Center	Study Design	Patient (n)	Train Image(n)	Diagnostician	Sensitivity (%)	Specificity (%)
Mu 2021 [37]	China	WLI	UNet++ ResNet	Multi	Prospective	4,587	7,326	AI	89	95
								Endoscopist	89	96
Lin 2021 [38]	China	WLI	TResNet	Multi	Retrospective	2,741	6,489	AI	97.9	97.5
								Endoscopist	42	96
Xu 2021 [39]	China	WLI ME-NBI BLI	VGG-16	Multi	Prospective	1,384	4,138	AI	90.1	86.1
								Endoscopist	77.8	76.5
Yang 2022 [40]	China	WLI LCI	SE-ResNet	Single	Retrospective	630	17,137	AI	96.6	97.9
Yan 2020 [41]	Macau	NBI ME-NBI	EfficientNet	Single	Retrospective	416	1,880	AI	91.9	86.0
								Endoscopist	86.5	81.4
Siripoppohn 2022 [42]	Thailand	WLI NBI	Improved BiSeNet	Single	Prospective	136	642	AI	93.13	80.0
Huang 2004 [43]	Taiwan	WLI	Customized Neural Networks	Single	Retrospective	104	84	AI	83.3	91.9
Li 2021 [44]	Macau	NBI	Improved ResNet	Single	Retrospective	242	840	AI	93.16	87.1
Wong 2022 [45]	Macau	ME-NBI	Improved ResNet	Single	Retrospective	420	1372	AI	93.6	91.2
								Endoscopist	86.5	81.4
Lai 2022 [46]	Macau	WLI NBI ME-NBI	Customized Neural Networks	Single	Retrospective	513	792	AI	96.1	88.42
Li 2023 [47]	China	ESE	Customized Neural Networks	Single	Retrospective	NA	837	AI	94.39	91.81
Pornvora-phat 2023 [48]	Thailand	WLI NBI	Improved BiSeNet	Single	Retrospective	NA	1599	AI	91	96

WLI: White light imaging; LCI: Linked-color imaging; NBI, Narrow-Band imaging; ME-NBI: Magnifying endoscopy with NBI; BLI: Blue-laser imaging; ESE: Electronic staining endoscopy.

### Study characteristics

Basic information of the 12 studies are shown in Table 1, and the participant characteristics of each study are shown in S3 Table. Among the 12 studies, three were prospective [37, 39, 42], and nine were retrospective; three studies [37–39] were multi-center and nine were single-center; three studies [44, 46, 47] used expert consensus as the diagnostic criterion and nine used pathological findings as the diagnostic criterion; three studies [37, 38, 43] used only plain white light imaging (WLI) model, and nine studies involved narrow-band imaging (NBI), magnified endoscopy with NBI (ME-NBI), blue laser imaging, and linked color imaging (LCI) model.

Mu et al. [37] developed a computer-aided system to identify non-gastritis, common gastritis, AG, and GIM. The system contains five deep learning models. ResNet was used for lesion classification and UNet++ network was used for lesion segmentation. We extracted only the diagnostic data for GIM.

Lin et al. [38] collected 7,037 WLI images and corresponding biopsy information from 14 hospitals. The images were classified into three categories: AG, non-AG, and GIM, based on pathological findings. The AI algorithm was TResNet.The sensitivity and specificity of the AI model to diagnose GIM were 97.9% and 97.5%.

Xu et al. [39] collected WLI, ME-NBI, and blue laser imaging (BLI) images from five hospitals for model training to identify AG and GIM. The models were tested on internal, external, and prospective video test sets. The diagnostic data were collected from a randomly selected prospective video test set.

Yang et al. [40] constructed a dataset containing 21,420 WLI and LCI images to train a AI model for recognizing AG and GIM. The authors propose a dual transfer learning strategy to improve the model’s performanc. We extracted the data of the AI model on the WLI-independent and LCI-independent test sets and then combined them.

Yan et al. [41] collected 2,357 NBI and ME-NBI images from 416 patients for training an AI model for recognizing GIM. The sensitivity, specificity, and accuracy of the model were 91.9%, 86.0%, and 88.8%, respectively. Although the AI models performed better than the human experts, there was no significant difference between them. We combined the test results of the AI model on the NBI set and the ME-NBI set.

Siripoppohn et al. [42] implemented semantic segmentation of GIM by adding three additional preprocessing techniques to the BiSeNet network and compared it with the classical semantic segmentation algorithms, DeepLabV3+ and U-Net. Diagnostic data were extracted from the improved algorithm using a prospective video test set.

Huang et al. [43] constructed custom neural networks for identifying lesions, such as H. pylori infection, atrophy, and GIM, and extracted data relevant to identifying GIM. Although the study prospectively selected 104 patients, the model was trained and tested based on the images of these patients; therefore, we considered this to be a retrospective study.

Li et al. [44] proposed a novel multi-feature fusion method to identify GIM, which extracts features from pixels, colors, and textures of endoscopic images, respectively. The authors trained and tested the model using 1,050 images and achieved an accuracy of 90.28%.

Wong et al. [45] proposed a novel broad-learning system stacking framework with multiscale attention. This method could reliably diagnose GIM with an accuracy of 93.2%. The authors also compared the AI model’s diagnostic capability to that of endoscopists, and the result showed that AI was competitive with that of skilled endoscopists.

Lai et al. [46] proposed a multi-scale multi-instance multi-feature joint-learning broad network. The network considers multiple features of each instance at multiple scales, resulting in more accurate classification. By training on a limited dataset, the network recognizes GIM with an accuracy of 85%.

Li et al. [47] proposed a combination of conventional and deep learning methods for IM lesion area localization and offset generation. The method could recognize the severity of GIM with an accuracy of 84.17%.

Pornvoraphat et al. [48] utilized AI techniques to achieve real-time segmentation of GIM. The AI algorithm is based on BiSeNet, and the authors used techniques such as negative sampling and label smoothing to improve the model’s performance. The sensitivity, specificity and accuracy of the AI model were 91%, 96% and 96%, respectively.

### Quality assessment

**Fig 2:**
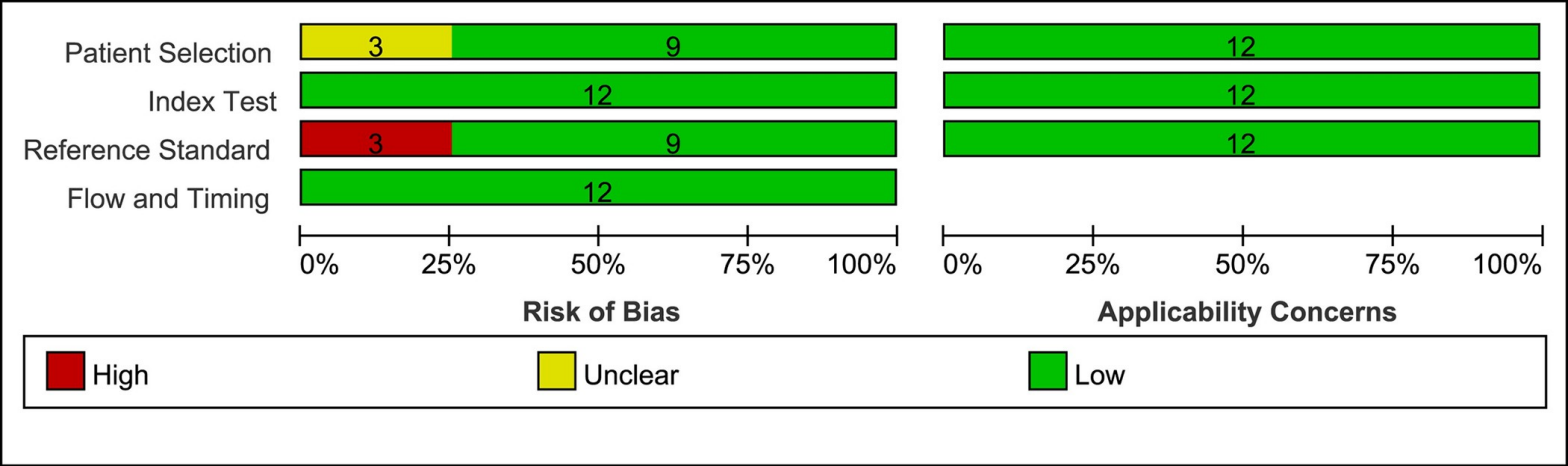
Results of the assessment of risk of bias in the included studies.

The quality was assessed using the Supplemented QUADAS-2 tool (Fig 2). In the patient selection section, three studies were of unknown risk. One study [40] did not state the source of the patients, while two studies [47, 48] did not state the number of patients included. In the reference standards section, three studies [44, 46, 47] used expert consensus rather than pathological findings as the gold standard and were considered high risk.

### Meta-analysis results

**Fig 3:**
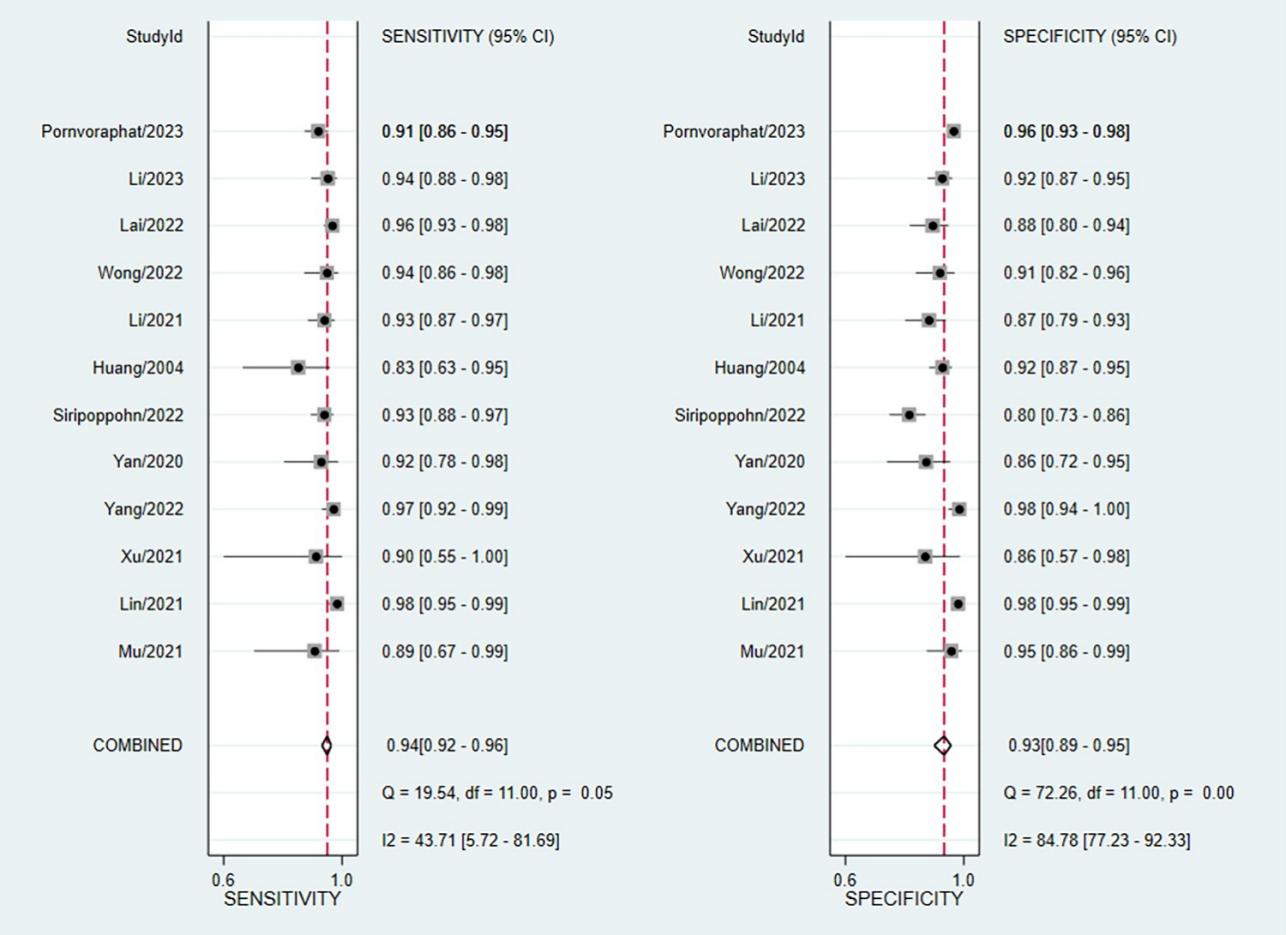
Forest plot of sensitivity and specificity.

**Fig 4:**
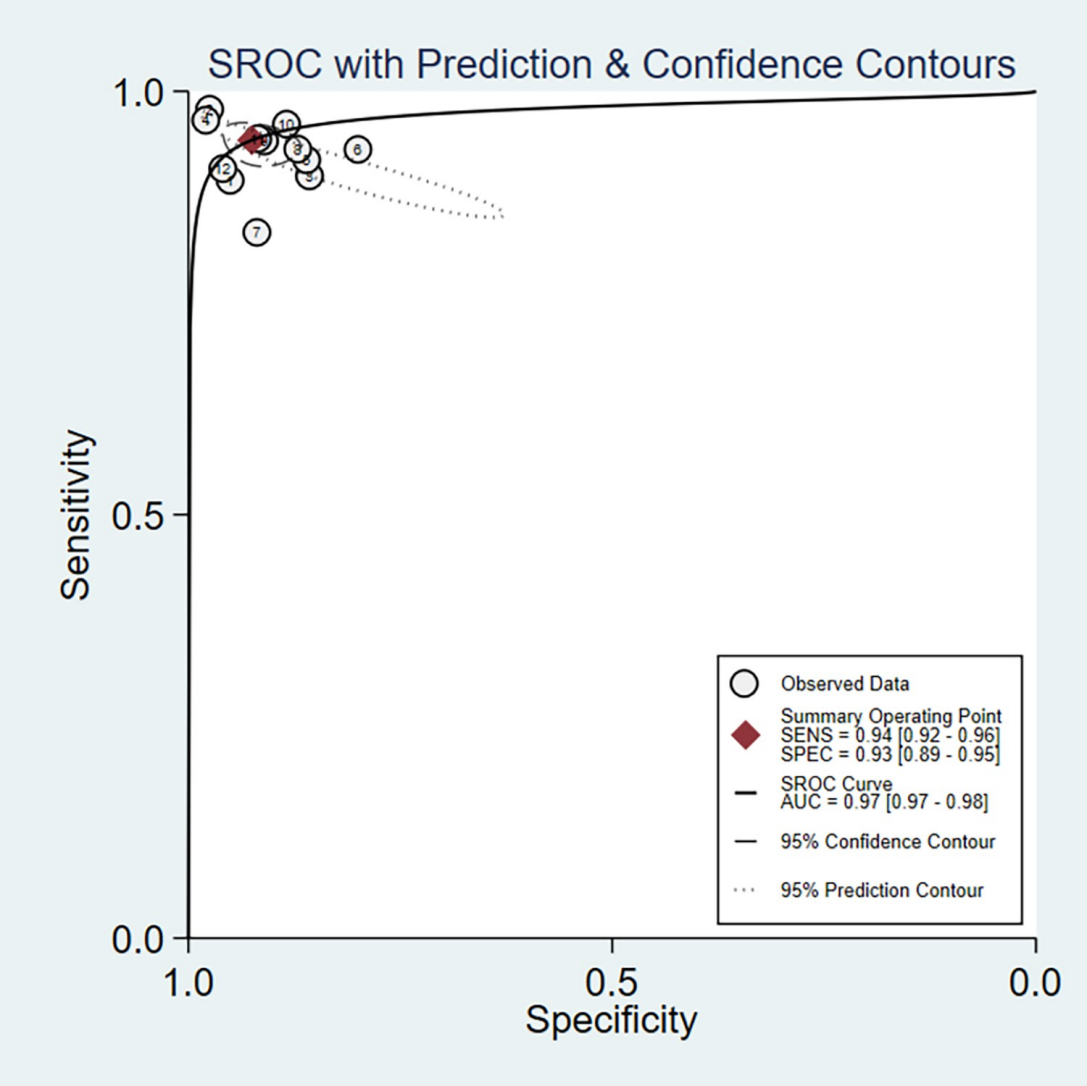
SROC curves.

We imported data from 12 studies into Stata/SE 16.0 for meta-analysis. The pooled sensitivity and specificity of AI diagnosing GIM were 94% (95% CI: 0.92–0.96, I^2^ = 43.71%) and 93% (95% CI: 0.89–0.95, I^2^ = 84.78%), respectively (Fig 3). The PLR and NLR were 12.59 (95% CI: 0.38–18.91) and 0.06 (95% CI: 0.05–0.09), respectively (S1 Fig). The DOR (S2 Fig) and AUC (Fig 4) were 201.5 (95% CI: 110.18–368.51) and 0.97 (95% CI: 0.97–0.98), respectively. With a PLR (12.59) greater than 10, it suggested that AI had the capability to confirm the diagnosis of GIM. The NLR value (0.06<0.1) indicates that AI can reliably exclude GIM. The DOR value (217>1) indicated a better discriminative effect of this diagnostic test, and an AUC (0.97) closer to 1 indicated a better diagnostic effect. It is important to note that the I^2^ of combined sensitivity and specificity suggest a high degree of heterogeneity between studies.

**Fig 5:**
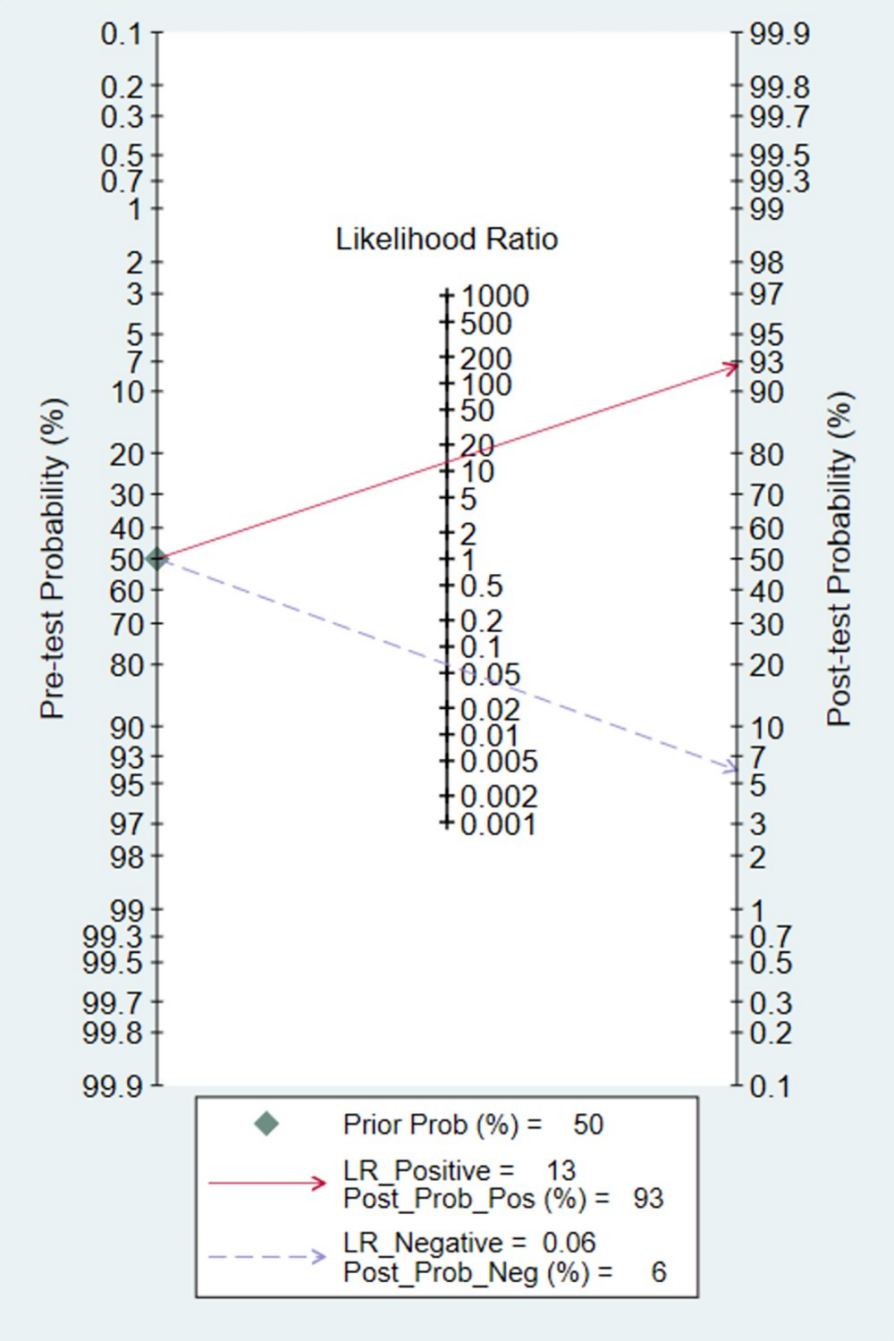
Fagan’s nomogram.

Fagan plots were drawn to evaluate the clinical applicability of AI (Fig 5). With a pre-test probability set at 0.5, a positive AI diagnostic result indicated a 93% probability of the patient having GIM, while a negative result suggested a 6% likelihood, confirming or excluding the presence of Gastrointestinal Intestinal Metaplasia (GIM).

### Subgroup analysis

We conducted subgroup analyses to investigate the impact of various factors on the performance of (AI) in diagnosing GIM. The factors included study design (prospective or retrospective), study center (multi-center or single-center), endoscopy type (WLI only or others), number of training images (>1500 or <1500), and algorithm type (classification algorithm or others) (Table 2).

**Table 2:** Subgroup analyses results.

Subgroup	Condition	Studies(n)	Sensitivity(95%CI)	P	Specificity(95%CI)	P
Number of training images	> 1500	6	0.94(0.92–0.97)	<0.001	0.94(0.91–0.98)	<0.001
	< 1500	6	0.94(0.92–0.97)		0.89(0.83–0.95)	
Endoscopy type	WLI only	3	0.95(0.90–0.99)	<0.001	0.95(0.92–0.99)	0.05
	others	9	0.94(0.92–0.96)		0.90(0.86–0.94)	
Study design	prospective	3	0.93(0.88–0.98)	<0.001	0.87(0.78–0.97)	<0.001
	retrospective	9	0.94(0.93–0.96)		0.93(0.90–0.96)	
Study center	multi-center	3	0.97(0.94–0.99)	0.01	0.94(0.90–0.99)	0.18
	single-center	9	0.94(0.92–0.95)		0.91(0.87–0.95)	
Algorithm type	classification	9	0.95(0.94–0.97)	<0.001	0.92(0.88–0.96)	0.02
	others	3	0.92(0.89–0.95)		0.92(0.85–0.99)	

The effect of study center on sensitivity was statistically significant, and the effect of other grouping conditions on sensitivity was extremely significant. The algorithm type had a significant effect on specificity. All of the above factors could be potential sources of heterogeneity between studies.

### Publication bias and sensitivity analysis

**Fig 6:**
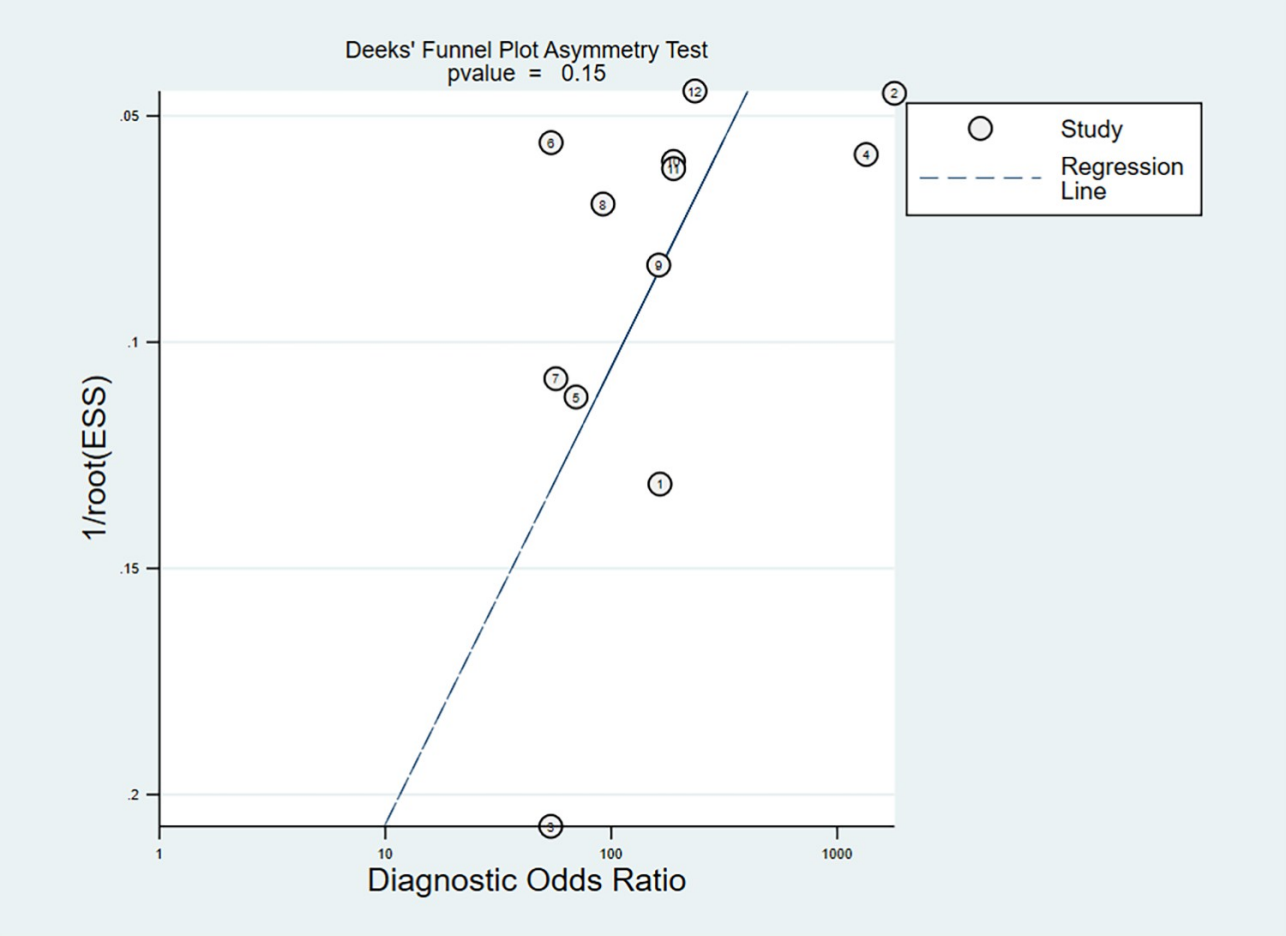
Deeks’ funnel plot asymmetry test for publication.

To assess the presence of publication bias, we performed a Deeks’ funnel plot asymmetry test (Fig 6). The P value was 0.15, indicating no significant publication bias.

To delve deeper into the heterogeneity among studies, we conducted a pooled analysis by systematically excluding each study one at a time. After removing the studies by Pornvoraphat [48], the most significant changes were observed in combined sensitivity and specificity, which were found to be 95% (95% CI: 0.93–0.96, I^2^ = 40.28%) and 92% (95% CI: 0.88–0.95, I^2^ = 83.04%), respectively. However, this is not significantly different from the original results, indicating that the meta-analysis results were relatively stable.

### AI vs. endoscopists

To further explore the diagnostic ability of the AI, we compared it to that of the endoscopists. We collected 5 sets of data from 12 studies (Table 1) for the meta-analysis. An essential criterion for data extraction was that the test sets used by the AI and endoscopists must be identical. The comparison results are shown in Table 3. Their specificities showed no significant disparity, and while AI exhibited a superior sensitivity compared to the endoscopists, this variance did not reach statistical significance.

**Table 3:** AI vs. endoscopists.

Performance Metrics	AI	Endoscopists	P
Sensitivity(95%CI)	0.95(0.91–0.99)	0.79(0.65–0.92)	0.39
Specificity(95%CI)	0.94(0.89–0.98)	0.90(0.83–0.96)	0.16

## Discussion

Dilaghi et al. [49] conducted a meta-analysis on AI’s role in the diagnosis of precancerous gastric lesions and *Helicobacter pylori* infection, with two studies involving the diagnosis of GIM. To the best of our knowledge, this is the first systematic review and meta-analysis focusing on the diagnosis of GIM using AI. This meta-analysis included 12 studies involving 11,173 relevant patients and 46,268 images/videos. In addition to pooling the diagnostic performance of AI, we explored the impact of factors such as different algorithms, varied image quantities, etc., on AI performance. Furthermore, we compared the diagnostic abilities of endoscopists with those of AI. The results demonstrate that various indicators of AI-assisted diagnosis for GIM exhibit satisfactory levels. This indicates that AI can help doctors diagnose GIM more accurately, thus reducing the rate of missed diagnoses and misdiagnoses. In addition, AI can accelerate the diagnostic process, which reduces doctors’ workload and improves their efficiency.

There are still some limitations of this study: (a) The diagnostic value of AI algorithms may not be adequately assessed due to the relatively small number of studies and limited sample size. (b) The 12 included studies were conducted in Asia, and the results of the meta-analysis may not apply to a wider population. (c) Heterogeneity among the studies was very high. Although subgroup analyses were conducted, the restricted number of studies did not allow for further analysis of influencing factors, such as the type of test set (image or video) and specific endoscope type (e.g., NBI or LCI). (d) Most studies have identified only intestinal metaplasia and atrophic gastritis, and further validation is needed to determine whether other lesions affect the determination of AI. (e) Most studies were retrospective, and the test set included static images. More prospective, real-time, endoscopic-video-based studies are required to validate whether AI can be adapted to complex endoscopic environments.

Among the twelve studies, one [47] employed AI to identify GIM and assess its severity through endoscopic image analysis. This process is crucial for accurately pinpointing the most representative lesion area for biopsy, indicating a significant avenue for future research. Additionally, identifying early malignant changes from GIM remains a challenge. Previous studies have used AI to identify early gastric cancer by analyzing WLI, NBI, or ME-NBI images [50–52]. Ikenoyama et al. [51] used conventional WLI images to identify early cancers smaller than 20mm, with AI sensitivity and specificity of 58.4% and 87.3%, respectively. While these results are encouraging, AI performance still needs to be improved.

Most included studies used deep learning techniques, but none explained AI’s decision-making process in detail. Due to their complexity and "black-box" nature, deep learning models often find it difficult to explain their internal working mechanisms and decision-making basis, which largely limits their clinical applications [53]. The introduction of algorithms such as LRP [54] and Grad-CAM [55] has played an important role in enhancing the explainability of existing deep learning models [56, 57]. Developing inherently explainable AI models will enable better application in clinical practice.

All the included studies were tested using their own datasets, and it was difficult to directly compare the performances of the models. High-quality publicly available datasets can be used as evaluation benchmarks to compare the performances of different algorithms. Additionally, publicly available datasets can encourage more people to participate in AI research. Currently, publicly available gastrointestinal image datasets include Hyper-Kvasir [58] and SUN-SEG [59]; however, there is a lack of large publicly available image datasets related to GIM.

It is worth noting that although AI applied to healthcare has made many technological breakthroughs, it also poses certain challenges to the current value system and legal system from the legal and ethical levels. For example, it may raise privacy and data security issues and legal liability issues when AI’s decisions are made incorrectly. As AI continues to advance, collaborative efforts among governments, healthcare organizations, and AI technology companies are crucial to establishing a robust framework that ensures the responsible and fal deployment of AI in clinical settings.

## Conclusions

The pooled sensitivity of our meta-analysis was 94% (95% confidence interval: 0.92–0.96) and specificity was 93% (95% confidence interval: 0.89–0.95). Comparisons by AI vs. endoscopists showed that AI had a higher sensitivity (95% vs. 79%). The results show that AI performed excellently in diagnosing GIM, which provides an evidence-based support for the clinical application of AI. At the same time, we identified some potential limitations, such as the quality of the dataset, generalizability of the AI model, and explainable AI. The application of AI-assisted endoscopy in the medical field is promising. Future research could focus on prospective studies, improvement of the explainability of models, and adaptation to different patient characteristics.

## Supporting information

S1 Table PRISMA-DTA checklist. (DOCX)

S2 Table Searching strategy to find relevant articles. (DOCX)

S3 Table Participant characteristics and algorithmic details of included studies. (DOCX)

S1 Fig Forest plot of PLR and NLR of AI in identifying GIM. (DOCX)

S2 Fig Forest plot for the diagnostic odds ratio and diagnostic score after combination. (DOCX)

## Abbreviations

AI: Artificial Intelligence
AUC: Area Under the SROC curve
BLI: Blue-Laser Imaging
CI: Confidence Interval
DOR: Diagnostic Odds Ratio
GIM: Gastric Intestinal Metaplasia
LCI: Linked-Color Imaging
ME-NBI: Magnifying Endoscopy with NBI
NBI: Narrow-Band Imaging
NLR: Negative Likelihood Ratio
PLR: Positive Likelihood Ratio
SROC: Summary Receiver Operating Characteristic
WLI: White-Light Imaging
