# Supplementary material for: Accuracy of artificial intelligence-assisted endoscopy in the diagnosis of gastric intestinal metaplasia: A systematic review and meta-analysis
Source: PLoS One. 2024 May 14;19(5):e0303421. doi: 10.1371/journal.pone.0303421 (PMC11093381; doi:10.1371/journal.pone.0303421)
Supplement: S2 Table — (DOCX) [file pone.0303421.s002.docx]

**S2 Table.** Searching strategy to find relevant articles.

| **Database: PubMed**  ((Deep Learning) OR (Artificial Intelligence) OR (Machine Learning) OR (Computer Aided Diagnosis) OR (neural networks) OR (Transformer)) AND ((Gastritis) OR (Gastric Precancerous) OR (Intestinal Metaplasia)) |
| --- |
| **Database: Embase**  ('artificial intelligence':ti,ab,kw OR 'deep learning':ti,ab,kw OR 'machine learning':ti,ab,kw OR 'computer aided diagnosis':ti,ab,kw OR 'neural networks':ti,ab,kw OR 'transformer':ti,ab,kw) AND ('gastritis':ti,ab,kw OR 'Gastric Precancerous':ti,ab,kw OR 'intestinal metaplasia':ti,ab,kw) |
| **Database: Cochrane Library**  #1 (Artificial Intelligence):ti,ab,kw OR (Deep Learning):ti,ab,kw OR (Machine Learning):ti,ab,kw OR (Computer Aided Diagnosis):ti,ab,kw OR (neural networks):ti,ab,kw  #2 (Transformer):ti,ab,kw  #3 #1 OR #2  #4 (Gastritis):ti,ab,kw OR (Gastric Precancerous):ti,ab,kw OR (Intestinal Metaplasia):ti,ab,kw  #5 #3 AND #4 |
| **Database: Web of Science**  (TS=(Artificial Intelligence) OR TS=(Deep Learning) OR TS=(Machine Learning) OR TS=(Computer Aided Diagnosis) OR TS=(neural networks) OR TS=(Transformer)) AND (TS=(Gastritis) OR TS=(Gastric Precancerous) OR TS=(Intestinal Metaplasia)) |
| **Database: IEEE Xplore**  ("All Metadata": "Artificial Intelligence" OR "All Metadata": "Deep Learning" OR "All Metadata": "Machine Learning" OR "All Metadata": "Computer Aided Diagnosis" OR "All Metadata": "neural networks" OR "All Metadata": "Transformer") AND ("All Metadata": "Gastritis" OR "All Metadata": "Gastric Precancerous" OR "All Metadata": "Intestinal Metaplasia") |
