# Supplementary material for: Accuracy of artificial intelligence-assisted endoscopy in the diagnosis of gastric intestinal metaplasia: A systematic review and meta-analysis
Source: PLoS One. 2024 May 14;19(5):e0303421. doi: 10.1371/journal.pone.0303421 (PMC11093381; doi:10.1371/journal.pone.0303421)
Supplement: S3 Table — (DOCX) [file pone.0303421.s003.docx]

**S3 Table.** Participant characteristics and algorithmic details of included studies.

| Study | Participant characteristics |
| --- | --- |
| Mu 2021 [37] | Internal test set  Male/Female 199 /187  Mean age, y (SD) 51.74 (11.48)  External test set  Male/Female 68/69  Mean age, y (SD) 53.54 (13.57)  Video test set  Male/Female 46 /34  Mean age, y (SD) 49.91 (12.93) |
| Lin 2021 [38] | Total number of patients 2,741  Male/Female 1387/1354  Mean age, y (SD) 52(13.2) |
| Xu 2021 [39] | Training set  Gastric atrophy  Male/Female 200/154  Mean age, y (SD) 58.1(9.2)  Intestinal metaplasia  Male/Female 209/170  Mean age, y (SD) 58.2(9.0)  Internal test set  Gastric atrophy  Male/Female 105/88  Mean age, y (SD) 57.3(10.0)  Intestinal metaplasia  Male/Female 96/72  Mean age, y (SD) 57.0(10.4)  External test set  Male/Female 111/102  Mean age, y (SD) 55.2(13.3)  Prospective video test set  Male/Female 43/34  Mean age, y (SD) 57.8(8.1) |
| Yang 2022 [40] | Training set and test set  Number of patients 600  Separate test set  Number of patients 30 |
| Yan 2020 [41] | Training set  Number of patients 336  Mean age (range) 48(23-96)  Male/Female 151/185  Test set  Number of patients 80  Mean age (range) 49(25-92)  Male/Female 38/42 |
| Siripoppohn 2022 [42] | Total number of patients 136 |
| Huang 2004 [43] | Total number of patients 104 |
| Li 2021 [44] | Total number of patients 242 |
| Wong 2022 [45] | Total number of patients 420 |
| Lai 2022 [46] | Total number of patients 234 |
| Li 2023 [47] | Null |
| Pornvoraphat 2023 [48] | Null |
