# Supplementary figures and images for: Accuracy of artificial intelligence-assisted endoscopy in the diagnosis of gastric intestinal metaplasia: A systematic review and meta-analysis
Source: PLoS One. 2024 May 14;19(5):e0303421. doi: 10.1371/journal.pone.0303421 (PMC11093381; doi:10.1371/journal.pone.0303421)

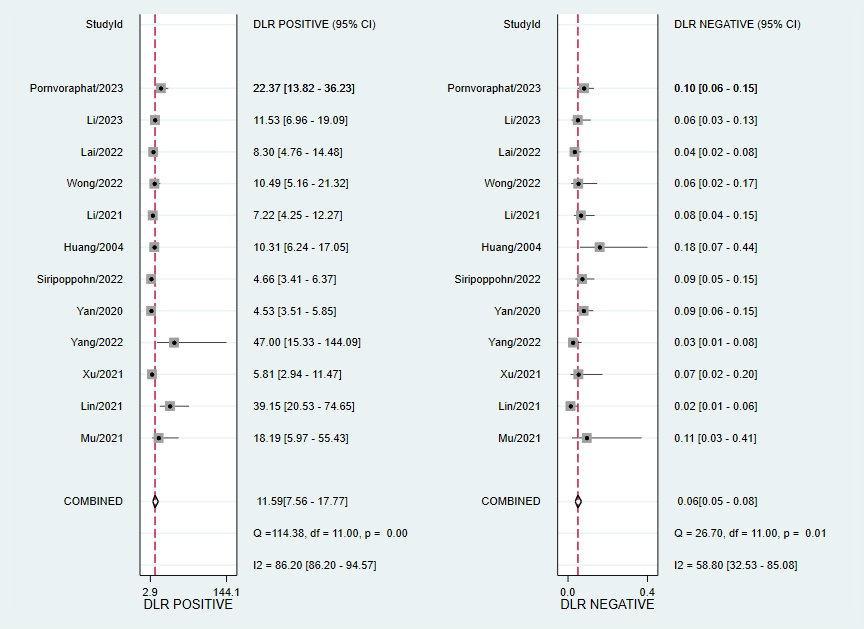


**S1 Fig.** Forest plot of PLR and NLR of AI in identifying GIM.

Supplement: S1 Fig — (DOCX) [file pone.0303421.s004.docx]

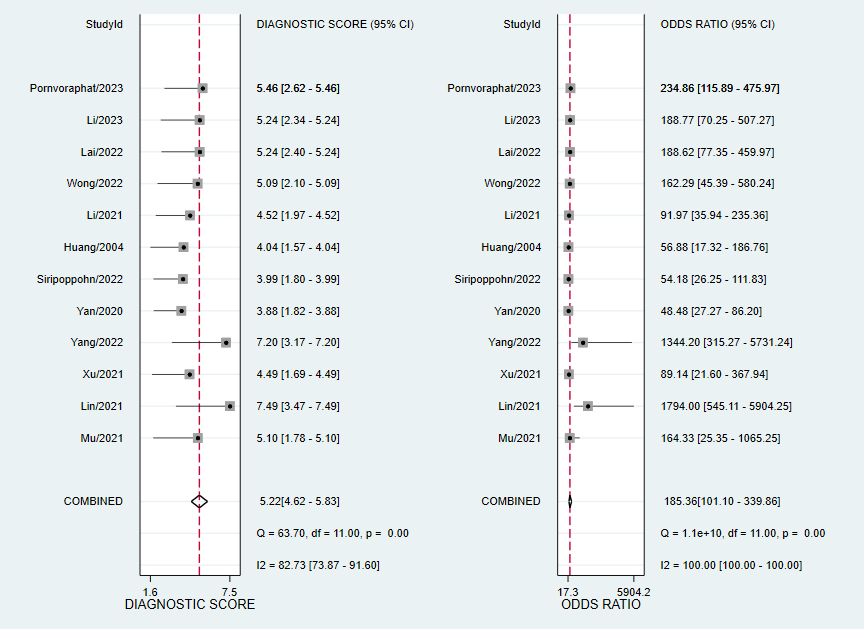


**S2 Fig.** Forest plot for the diagnostic odds ratio and diagnostic score after combination.

Supplement: S2 Fig — (DOCX) [file pone.0303421.s005.docx]
